# Fast fragment- and compound-screening pipeline at the Swiss Light Source

**DOI:** 10.1107/S2059798322000705

**Published:** 2022-02-21

**Authors:** Jakub W. Kaminski, Laura Vera, Dennis P. Stegmann, Jonatan Vering, Deniz Eris, Kate M. L. Smith, Chia-Ying Huang, Nathalie Meier, Julia Steuber, Meitian Wang, Günter Fritz, Justyna A. Wojdyla, May E. Sharpe

**Affiliations:** a Swiss Light Source, Paul Scherrer Institute, 5232 Villigen PSI, Switzerland; b Department of Cellular Microbiology, Institute of Biology, University of Hohenheim, 70599 Stuttgart, Germany

**Keywords:** fragment-based drug discovery, protein crystallography, automation, high throughput, software, FFCS

## Abstract

Over the last two decades, fragment-based drug discovery (FBDD) has emerged as an effective and efficient method to identify new chemical scaffolds for the development of lead compounds. X-ray crystallography can be used in FBDD as a tool to validate and develop fragments identified as binders by other methods. However, it is also often used with great success as a primary screening technique. In recent years, technological advances at macromolecular crystallo­graphy beamlines in terms of instrumentation, beam intensity and robotics have enabled the development of dedicated platforms at synchrotron sources for FBDD using X-ray crystallography. Here, the development of the Fast Fragment and Compound Screening (FFCS) platform, an integrated next-generation pipeline for crystal soaking, handling and data collection which allows crystallography-based screening of protein crystals against hundreds of fragments and compounds, at the Swiss Light Source is reported.

## Abstract 2

The FFCS platform has recently been developed by the Swiss Light Source macromolecular group. It is an integrated next-generation X-ray crystallography fragment-based drug-discovery pipeline for crystal soaking, handling and data collection.

## 1. Introduction

The identification of small molecules that modulate protein function and activity is a crucial step in drug development. Fragment-based drug discovery (FBDD) has emerged as an effective and efficient method to identify promising chemical scaffolds for the development of lead compounds (Erlanson *et al.*, 2016 ▸). FBDD is an alternative to high-throughput screening (HTS), which uses biochemical or biophysical assays to search for lead compounds within large libraries that have tens of thousands to millions of members (Fox *et al.*, 2006 ▸). The size of these libraries presents significant logistical challenges, but they can still sample only a small portion of the possible combinatorial chemical space (Hann & Oprea, 2004 ▸) and are frequently expanded to address new targets. HTS is effective in identifying compounds that bind tightly to target proteins, but these molecules are often not drug-like due to high lipophilicity or disadvantageous pharmacokinetic properties. From an imperfect and over-elaborate starting point, optimizing (or retaining) the potency and selectivity of hits while simultaneously improving bioavailability can be extremely challenging. In the past two decades, these inherent difficulties of HTS have triggered the development of FBDD, which has attracted increasing interest from both academia and industry (Erlanson *et al.*, 2016 ▸).

In contrast to HTS, FBDD utilizes small libraries (1000–3000 members) of simple molecules (<300 Da in molar mass, *c*log*P* <3, <3 rotatable bonds, <3 hydrogen-bond donors or acceptors; Congreve *et al.*, 2003 ▸). For simple molecules, a small library can effectively sample the respective chemical space. Fragments typically bind weakly to target proteins (*K*_d_ = 10^−2^–10^−4^ *M*) but with high ligand efficiency. In order to progress towards high-affinity compounds, the binding affinity of hits can be increased by coupling fragments (‘linking and merging’) or derivatizing a single fragment (‘growing’). This process affords a greater understanding and control of the binding mode and pharmacokinetic properties of the resulting lead compounds compared with the development of HTS hits. To date, drug candidates arising from FBDD programmes have achieved considerable success, with six drugs approved by the United States Food and Drug Administration (USFDA) and more than 15 candidates currently in clinical trials (Erlanson *et al.*, 2016 ▸; Jahnke *et al.*, 2020 ▸; http://practicalfragments.blogspot.com/2021/11/fragments-in-clinic-2021-edition.html).

The detection of binding during fragment-library screening is confined to assays with appreciable throughput that can detect binding with dissociation constants in the high-micromolar to millimolar range. In these assays, high concentrations of fragments must be used to achieve an appreciable saturation of binding, necessitating the use of co-solvents (usually DMSO) to increase solubility. Commonly used techniques for fragment screening include surface plasmon resonance, nuclear magnetic resonance, thermal denaturation midpoint-shift assays and mass spectrometry. When evaluating the development potential of fragment hits, other lower-throughput techniques such as isothermal titration calorimetry, microscale thermophoresis, biochemical assays and X-ray crystallography can be used to provide information about binding affinity and thermodynamics, as well as binding location and orientation.

In addition to its use in validating and characterizing the binding of fragment hits identified by other techniques, X-ray crystallography has achieved great success as a primary screening method. It is one of the most sensitive and reliable techniques for the detection of binding, and gives key structural information on fragment poise. In addition, X-ray crystallographic FBDD (xFBDD) can be ‘site-agnostic’, identifying and locating fragments bound both at known active sites and at allosteric sites. Thus, it allows druggability to be assessed for the whole accessible protein surface, without assumptions, and in the absence of known high-affinity binders. The use of X-ray crystallography for primary screening in FBDD was first demonstrated in 1997 (Verlinde *et al.*, 1997 ▸). Since then, the clear advantages of the method have established it as an integral part of the drug-discovery platforms in several companies (Spurlino, 2011 ▸; Hubbard *et al.*, 2007 ▸; Davies *et al.*, 2006 ▸; Price *et al.*, 2017 ▸). In the last half-decade, the approach has become more widely adopted. Technological advances at MX beamlines in terms of instrumentation, beam intensity and robotics have enabled the development of dedicated screening platforms at synchrotron sources. This approach was spearheaded by XChem at Diamond Light Source (DLS), which offered the first proposal-based full xFBDD (hundreds of fragments) platform to synchrotron users (Douangamath *et al.*, 2021 ▸; Krojer *et al.*, 2017 ▸). In parallel or subsequently, xFBDD platforms were developed at other synchrotron sources (Wollenhaupt *et al.*, 2021 ▸; Lima *et al.*, 2021 ▸; Cipriani *et al.*, 2012 ▸; Lima *et al.*, 2020 ▸).

The Swiss Light Source (SLS) macromolecular crystallo­graphy (MX) group has recently developed their own xFBDD platform, called the Fast Fragment and Compound Screening (FFCS) platform. In this publication, we present this newly established FFCS pipeline, focusing on hardware and software solutions.

## 2. Outline of the FFCS pipeline

**Figure 1:**
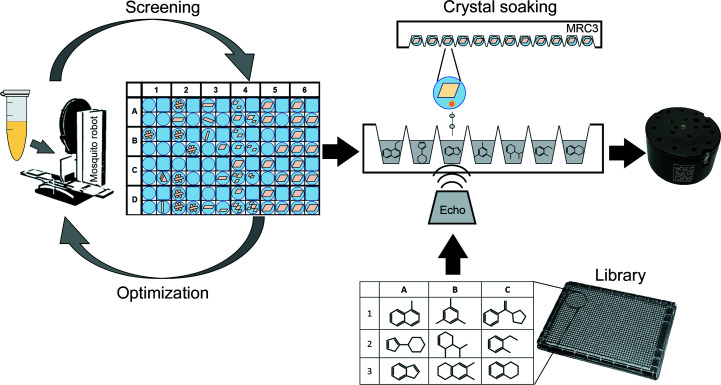
Schematic of the FFCS pipeline. Initial crystals are optimized in SWISSCI MRC-3 plates and tested for DMSO resilience. Plates are stored in Rock Imager systems at 4°C or 20°C and are imaged at regular intervals. Selected fragments are contactlessly transferred from small-volume stock solutions to preselected crystal drops with the Echo liquid-handling device. Subsequent semi-automated crystal harvesting, supported by the Crystal Shifter robot, allows the efficient freezing of soaked crystals.

The FFCS pipeline was developed within the MX group at the SLS. The main aim of the FFCS project was the development of an integrated next-generation pipeline for crystal soaking, handling, and data collection and analysis, which would aid high-throughput drug-discovery processes in the pharmaceutical industry and academic laboratories. The FFCS pipeline includes the following steps (Fig. 1 ▸).

(i) Crystal growth: crystals are grown in SWISSCI MRC-3 plates, which are stored and imaged in a Rock Imager (Formulatrix) at 4°C or room temperature (RT).
(ii) Crystal soaking: performed with an Echo 550 (Collins *et al.*, 2017 ▸).
(iii) Crystal harvesting: assisted by a Crystal Shifter robot (Oxford Lab Technologies; Wright *et al.*, 2021 ▸).
(iv) Data collection: at one of the three SLS MX beamlines.
(v) Initial data processing and structure solution: within the beamline computing environment.

The process, which involves handling hundreds of crystals and fragments/compounds, requires the creation of tools to make it faster, more efficient and more automated. The crucial components of the FFCS pipeline are the hardware setup and the dedicated in-house-developed software, the *FFCS* software suite, which allows easy experiment design, efficient bookkeeping and links all of the steps of the FFCS pipeline. Close collaboration with the group of Günter Fritz, University of Hohenheim, enabled the extension of the initial data processing and structure solution with an advanced data-evaluation pipeline developed in his group (Stegmann *et al.*, in preparation).

## 3. Hardware setup

In order to accommodate new equipment, the SLS Macromolecular Crystallization Facility (CF), which is localized in close proximity to the MX beamlines, was rearranged substantially. A separate space dedicated solely to the FFCS equipment was created and adapted accordingly. The FFCS hardware setup was to a large extent inspired by the XChem Fragment Screening facility at DLS, which is the largest facility for high-throughput crystallographic fragment screening that is tailored for academic users.

The Crystallization Facility is equipped for robotic crystallization screening using sitting-drop vapour diffusion (or lipidic cubic phase) with a Mosquito robot (SPT Labtech), which is available at both 4°C and RT. Crystallization plates are barcoded, stored and imaged in dedicated 4°C or RT Rock Imager (Formulatrix) plate hotels. The Echo 550 noncontact liquid handler (Labcyte) is mounted on a specialized vibration-free table to prevent any interference during acoustic droplet injection experiments. To ensure longevity and to maintain high quality, FFCS fragment and compound libraries are stored in barcoded Echo Qualified 384-well or 1536-well COC plates (Labcyte) in a dedicated storage system. The Storage­Pod system (Roylan Developments) consists of storage pods housing the fragment libraries and a controller unit to purge the storage pods with dry nitrogen gas. This enables the storage of fragment and compound libraries in an inert, moisture-free and oxygen-free environment, preventing their dilution, precipitation and damage. The library storage plates are stored at room temperature, since repeated freeze–thaw cycles of DMSO-dissolved substances are associated with compound degradation (Kozikowski *et al.*, 2003 ▸). To further extend their durability, the FFCS library storage plates are heat-sealed with a PlateLoc Thermal Microplate Sealer (Agilent) using an aluminium-based material seal.

The harvesting of fragment/compound-soaked crystals is assisted by a Crystal Shifter (CS) robot (Oxford Lab Technologies). The CS device is a motorized *xy* microscope stage with computer software which enables semi-automated error-reduced crystal picking by hand while preventing drying of the crystallization drops. The harvesting station is also equipped with a puck scanning station (the 3D-printing design of which was kindly shared with us by the DLS staff) equipped with two dedicated video cameras which enable scanning of both the puck and, optionally, the pin barcodes.

An extended effort was put into the FFCS laboratory design to ensure the ergonomic use of space and to guarantee an optimal workflow. Devices are located in close proximity in an arrangement reflecting the steps in the experimental procedure. Solutions such as a liquid-handler vibration-free table and a dedicated library storage system bestow robustness and reliability to the pipeline and improve upon the already existing facilities.

## 4. *FFCS* software suite

**Figure 2:**
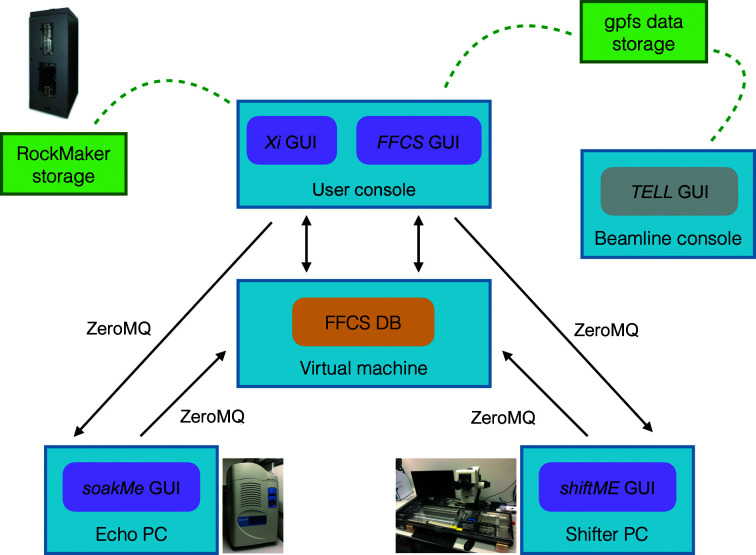
Schematic representation of the *FFCS* software suite. The user console allows the design and control of experiments *via* the *FFCS* GUI. Relevant experimental metadata are stored in the dedicated FFCS DB database and transferred directly *via* the network to Echo and Crystal Shifter controller PCs. Rock Maker and central general parallel file system (gpfs) data storages (shown in green) are mounted on the user console; gpfs data storage is also mounted on the beamline console.

The in-house-developed *FFCS* software suite facilitates experiment design and seamless step-by-step progression within the FFCS pipeline while efficiently record-keeping on behalf of the user. The *FFCS* suite consists of the FFCS database (FFCS DB) and four graphical interfaces: the *FFCS* GUI, *Xi* GUI, *soakedMe* GUI and *shiftMe* GUI (Fig. 2 ▸). A dedicated experimental FFCS user console (Red Hat Enterprise Linux 7 OS) as well as the Echo PC (Windows 10 OS) and the Crystal Shifter PC (Windows OS) are all located within the crystallization facility laboratory and are part of the same network. The user designs experiments in the *FFCS* graphical user interface (GUI) and picks crystals for soaking in the *Xi* GUI; these are accessible on the user console (remote access is also possible in the form of a full graphical user session via *NoMachine*; https://www.nomachine.com/). The *FFCS* GUI sends input files for Echo and Crystal Shifter experiments, while the *soakedMe* (Echo PC) and *shiftMe* (Crystal Shifter PC) GUIs report the results of soaking and fishing experiments back to the *FFCS* GUI.

### 4.1. FFCS database and *FFCS* GUI

The FFCS DB system receives and stores all of the metadata produced by users throughout FFCS campaigns. The FFCS DB is deployed on a dedicated virtual machine (Linux OS) as a docker container (https://www.docker.com/). The FFCS DB benefits from a MongoDB (https://www.mongodb.com/) schema-less design, which does not enforce a strict format of metadata. Currently implemented FFCS DB collections are ‘Plates’, ‘Wells’ and ‘Libraries’. Each collection includes an implemented validator to check for required keywords for each insert operation. Moreover, operation is not permitted if one of the keywords does not exist or the keywords are of the wrong type. All Python software that needs access to data in the FFCS DB utilizes a dedicated client, which is tailored to retrieve specific data as required by the database.

The *FFCS* GUI is a frontend PyQt5 (Python 3.6) application developed to interact with documents stored in the FFCS DB. It can be accessed via a dedicated FFCS user console that utilizes the same group accounts (nonpersonal e-accounts) to authenticate users at the beamline consoles. This allows the storage of all FFCS project-related documents in the same working space as is used for X-ray diffraction data collection. The e-account FFCS folder includes (i) copies of the crystallization drop images, (ii) Echo and Crystal Shifter input files (in .csv format), (iii) a data collection spreadsheet compatible with the *TELL* GUI, user software which controls the TELL automatic sample changer (Martiel *et al.*, 2020 ▸), (iv) the results of the automated data-processing pipelines (*adp* and *dimmer*), (v) a spreadsheet with FFCS experiment reports and (vi) fragment-library file(s) (in .sdf format).

**Figure 3:**
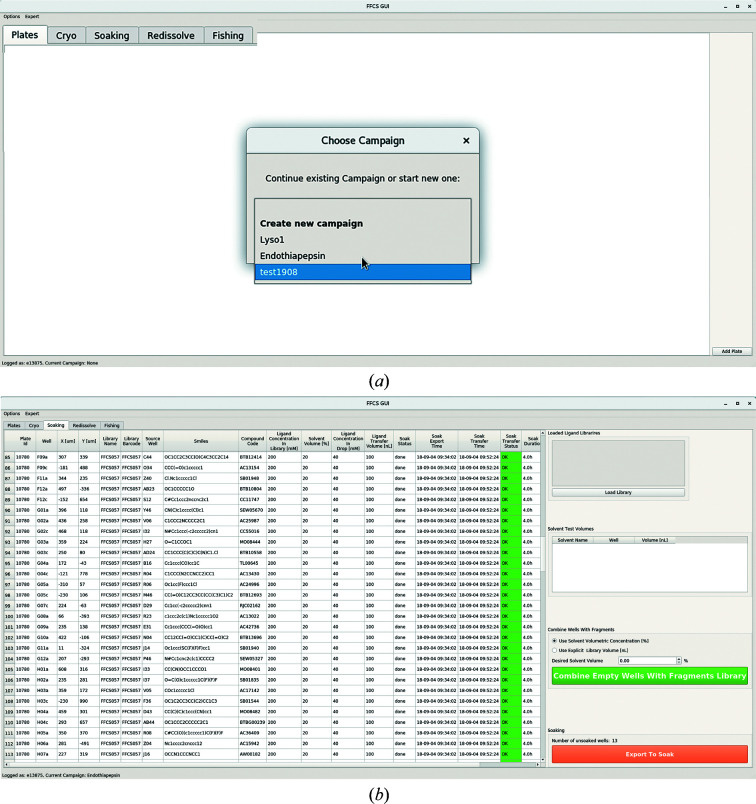
The *FFCS* GUI. (*a*) The startup view with a central popup message. Tabs representing steps of the FFCS campaign are enlarged for clarity. (*b*) The Soaking tab showing the status of a fragment-soaking step; the example here is from the FFCS endothiapepsin campaign.

Upon startup of the *FFCS* GUI, the user is presented with a menu listing all ongoing FFCS campaigns associated with a given e-account (the campaign name functions as a unique identifier); alternatively, users can start a new campaign (Fig. 3 ▸ *a*). The *FFCS* GUI tabs represent consecutive stages of a given campaign, namely ‘Plates’, ‘Cryo’, ‘Soaking’, ‘Redissolve’ and ‘Fishing’. Crystallization plates can be added to the ongoing FFCS campaign in the Plates tab. The plate ID can be typed in manually or it can be scanned with the barcode reader attached to the user console. The list of crystallization plates associated with the given FFCS campaign is retrieved by the *Xi* GUI and updated after the dispensing locations have been selected. The Cryo tab is an optional step of the FFCS pipeline and must be performed before the final soaking experiment. The Soaking tab allows the selected fragments list to be combined with the targeted crystallization drops (Fig. 3 ▸ *b*). The ‘Export to Soak’ button triggers ZeroMQ (https://zeromq.org/) transfer of the soaking-definition file to the Echo PC and of the Fishing definition file to the Crystal Shifter PC. It also starts a soaking duration timer, which is automatically refreshed every minute in the ‘Soak Duration’ column. After soaking has been completed, the Echo experiment report is sent to the FFCS DB, the Soaking tab timer is stopped, and the soaking status displayed in the *FFCS* GUI is updated accordingly. The Redissolve tab is an optional step of the FFCS pipeline which was created on demand for a challenging project that required DMSO-free soaking (Bedi *et al.*, 2020 ▸). The final Fishing tab displays the results of crystal harvesting with the Crystal Shifter robot and includes an ‘Export Data Collection Excel Sheet’ button, which enables the export of a data-collection sample spreadsheet that is compatible with the SLS data-acquisition software.

### 4.2. *Xi* GUI

**Figure 4:**
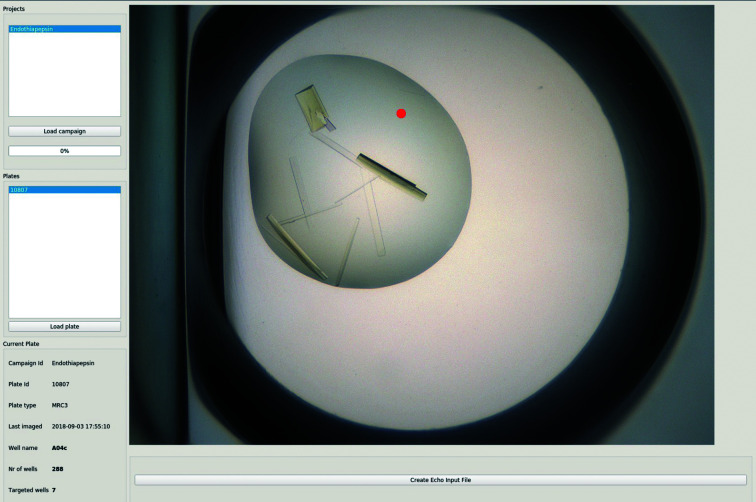
The *Xi* GUI showing an endothiapepsin crystallization drop. The red dot indicates the position selected for targeted acoustic delivery of a fragment dissolved in DMSO.

The *Xi* GUI is a PyQt5 interface (Python 3.6) which allows the easy identification of drops with suitable crystals and the precise definition of the fragment-dispensing location for targeted soaking experiments. Upon startup the GUI (i) connects to the FFCS DB and identifies FFCS campaigns for a given user (based on the e-account name), (ii) extracts the barcodes of the crystallization plates assigned to a selected campaign and (iii) copies images from the Rock Maker storage to the dedicated FFCS campaign folder. Images of crystallization drops are displayed in the central *Xi* GUI window and the dispensing location can be assigned with a single mouse click (Fig. 4 ▸). Dispensing locations can be added/removed with left/right mouse clicks, while moving to the next/previous image is controlled with the left/right keyboard arrows. The total number of wells for a given plate, as well as the number of targeted wells, are displayed in the bottom left corner of the GUI. Selected well locations are exported to the FFCS DB as a list of *x*, *y* coordinates, which are displayed in the *FFCS* GUI Soaking tab.

### 4.3. ZeroMQ communication

We created a suite of ZeroMQ servers and clients and two dedicated GUIs to enable the easy transfer of files between computers within the FFCS pipeline. As mentioned in the previous section, upon user request the *FFCS* GUI sends input files to the Echo and Crystal Shifter PCs. These input files contain sufficient information for the FFCS Soaking and Fishing pipeline steps without disclosing any confidential or user-specific information. After completion of the soaking/fishing steps, dedicated GUIs (*soakedMe* and *shiftMe*) assist users in sending results back to the FFCS DB. The *soakedMe* GUI (Echo PC) sends an Echo dispensing status report, which includes error messages. The *shiftMe* GUI (Crystal Shifter PC) combines results from Crystal Shifter fishing and the barcode reading station before sending the report back. Both soaking and fishing experiment results are displayed for user inspection in the *FFCS* GUI.

## 5. Data collection and initial analysis

The X-ray diffraction data are collected manually or automatically on one of the three SLS MX beamlines (PXI X06SA, PXII X10SA and PXIII X06DA). Manual data collection is performed using the SLS MX *DA*+ data-acquisition (daq) software GUIs, namely the *DA*+ GUI (Wojdyla *et al.*, 2018 ▸) and the *TELL* GUI, which controls the TELL automatic sample changer (Martiel *et al.*, 2020 ▸). Alternatively, un­attended data collection with the *Smart Digital User* (*SDU*) automation software package is available at all three MX beamlines (Wojdyla *et al.*, in preparation). *SDU* is a software alternative to the user controlling the experiment with the *DA*+ GUI and is an integral part of the SLS MX distributed daq software stack, communicating with many other software instances such as the *TELL* GUI and the mxdb database. *SDU* data collection and processing options can be defined by users in a sample spreadsheet (the template can be downloaded from the SLS MX webpage); alternatively, beamline-specific default parameters are applied. The automatic data processing (*adp*) provides instantaneous feedback on a subset of collected data (the first ‘fast_xds’ step) as well as fully processed data for the total angular range. In the second ‘complete’ step users can select one of the following processing pipelines: the in-house-developed *gopy*, *autoPROC* (Vonrhein *et al.*, 2011 ▸) or *xia*2*dials* (Winter *et al.*, 2018 ▸). The sample spreadsheet includes a column to indicate the name of the molecular-replacement model (located in a dedicated folder) which is utilized in the *dimple *pipeline (Wojdyr *et al.*, 2013 ▸) run within dedicated *dimmer* daemon wrappers. In the case of crystals soaked using the FFCS pipeline, the *dimmer* daemon extracts SMILES (simplified molecular-input line-entry system) codes for the fragments from the FFCS DB and generates ligand coordinates and restraints using *eLBOW* from the *Phenix* suite (Moriarty *et al.*, 2009 ▸). The resulting *dimmer* output is saved in a dedicated FFCS folder (organized on a per-campaign and per-sample basis), easing further downstream data analysis.

## 6. FFCS implemented

**Figure 5:**
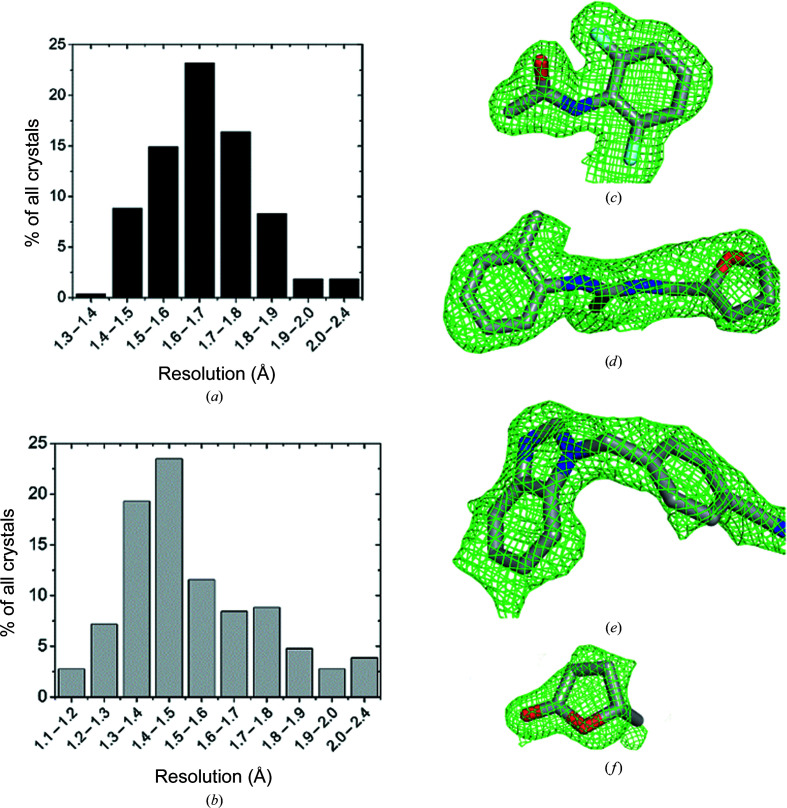
FFCS campaign results from two collaborative projects. (*a*) Resolution histogram of project 1 data sets shows that most crystals diffracted to around 1.7 Å (total range of 1.3–2.4 Å). (*b*) Resolution histogram of project 2 data sets shows that most crystals diffracted to 1.5 Å (total range of 1.1–2.4 Å). (*c*)–(*f*) Examples of bound fragments. Fragments were automatically placed by *FLYNN* (https://www.eyesopen.com/) into the *F*_o_ − *F*_c_ electron density from *dimple* runs. The *F*_o_ − *F*_c_ electron density is shown in green at 1.5σ. The fragments are (*c*) CD04232, *N*-(2,6-difluorophenyl)acetamide, PDB entry 7qu0, (*d*) BTB00030, 3-[(furan-2-yl)methyl]-1-(2-methylphenyl)thiourea, PDB entry 7qty, (*e*) Z26333434, 4-[(1*H*-1,3-benzodiazol-1-yl)methyl]benzonitrile, PDB entry 7qu3 and (*f*) AC14079, 5-methyloxolan-2-one, PDB entry 7qu5. Figures were generated automatically with *PyMOL* (https://pymol.org/) by the evaluation pipeline.

In order to evaluate the effectiveness and reliability of the FFCS pipeline under real-life conditions, we performed a set of in-house campaigns. Starting from initial tests with lysozyme and thermolysin, we progressed to endothiapepsin, the classic fragment-screening target (Köster *et al.*, 2011 ▸; Huschmann *et al.*, 2016 ▸; Schiebel *et al.*, 2016 ▸; Wollenhaupt *et al.*, 2020 ▸). One of the first academic collaboration FFCS projects was performed on protein crystals of two homologous bacterial enzymes. These systems functioned as challenging ‘real-life’ test cases for our pipeline. The two crystal systems relied on different precipitants, PEG and high salt, and both crystal systems had to be adapted by a distinct post-crystallization treatment to cope with the addition of large amounts of DMSO in the soaking procedure (Stegmann, Sharpe *et al.*, in preparation). For both projects, the 500 most diverse fragments (selected based on the structure descriptor FragFP) and 50 project-specific fragments were selected from one of the FFCS pipeline fragment libraries (Maybridge Library Ro3, Thermo Fisher). Once the soaking conditions had successfully been optimized, for each of the studied crystal systems two to three days of intermittent laboratory work were sufficient to perform soaking and crystal harvesting (∼250 crystals per day). The assisted harvesting with the Crystal Shifter robot resulted in an average of 50–60 crystals being fished per hour for both tested systems. These numbers may be higher for a trained user considering that both projects exhibited some skin formation on top of the crystallization droplets. The X-ray diffraction data sets were collected on beamlines PXI X06SA and PXIII X06DA either manually or with the *SDU* software. Both manual data collection and *SDU* resulted in 12–14 data sets with a full 360° rotation per hour. The resolution for diffraction data collected within the reported projects after optimization was 1.7 and 1.5 Å on average (resolution cutoff as defined by the *gopy* protocol in the *adp* software; Wojdyla *et al.*, 2018 ▸) for projects 1 and 2, respectively, exhibiting a rather narrow distribution across all data sets (Figs. 5 ▸ *a* and 5 ▸ *b*). Data sets processed with *adp* (with the in-house *XDS*-based *gopy* pipeline) were fed into *dimmer* workers which calculate *F*_o_ − *F*_c_ difference density maps and generate fragment coordinates. Subsequent steps of data analysis were performed using the data-evaluation pipeline developed in the group of Günter Fritz. In brief, the pipeline consists of the following steps: (i) the creation of chemical restraints for each fragment with *AceDRG* (Long *et al.*, 2017 ▸), (ii) automatic fitting of fragments into the difference density with *rhofit* (Smart *et al.*, 2014 ▸), (iii) post-refinement including the proper placement of side chains and water molecules with *REFMAC*5 (Murshudov *et al.*, 2011 ▸), *Coot* (Emsley *et al.*, 2010 ▸) and *phenix.refine* (Liebschner *et al.*, 2019 ▸), and (iv) estimation of the binding affinities of bound fragments by evaluation through a scoring function with *smina* (Koes *et al.*, 2013 ▸) and *SmOG* (Noel *et al.*, 2016 ▸). A bash script executing analysis-pipeline steps relies on a spreadsheet that contains the names of the data sets and the corresponding SMILES codes of the soaked fragments (input can be derived from the campaign spreadsheet exported from the *FFCS* GUI). An interactive helper script defines data source and destination paths and enables parallelization of the calculations. The entire fitting and evaluation method will be described and exemplified in detail elsewhere (Stegmann, Vering, Steuber & Fritz, in preparation). The hit rates for the two example projects were very similar, at 6.2% and 6.4%, respectively, and showed well defined, tightly bound fragments (Table 1 ▸).

## 7. Summary

With recent advances in MX automation and the consequent increase in popularity of xFBDD, we have implemented the FFCS pipeline at the SLS to offer users an additional avenue of using X-ray crystallography to generate and evaluate starting points for lead development in a rapid manner. Whereas the hardware setup for the FFCS pipeline is based on existing XChem solutions, the *FFCS* software suite was developed in-house. The SLS MX FFCS pipeline has been in operation since 2019, with a number of successful academic collaborations and industry customer projects. At the onset of the COVID-19 pandemic the project portfolio was expanded by a Severe acute respiratory syndrome coronavirus 2 (SARS-CoV-2) protein target (Sutanto *et al.*, 2021 ▸). Experience from the first FFCS campaigns clearly emphasized the importance of two crucial aspects of the pipeline: (i) DMSO optimization and (ii) software flexibility.

Before carrying out a large-scale soaking step, which can be performed with hundreds of fragments or compounds in a matter of hours, we first optimize the soaking conditions in order to obtain optimal data quality. Here, we vary the soaking time and concentration of the DMSO added to ascertain the appropriate soaking parameters. This often requires two to three rounds of iteration (a laboratory part combined with X-ray data collection). For crystal systems that require cryoprotection, additional rounds of testing are completed. Understanding the significance of the DMSO optimization step is necessary in order to perform a successful xFBDD campaign (Wollenhaupt *et al.*, 2021 ▸; Collins *et al.*, 2018 ▸), especially in the case of challenging crystallization systems such as the collaborative project described in the section above.

The advantage of the flexible in-house-developed *FFCS* software suite was clearly demonstrated in a collaborative project on the human YTHDC1 domain. DMSO optimization revealed that the active site is occupied by a tightly bound solvent molecule that prevents the binding of a positive control. Based on results from initial optimization we instead performed DMSO-free soaking, in which preselected fragments dissolved in DMSO were deposited onto a crystallization plate with the Echo device, air-dried to remove the solvent and redissolved in the crystallization buffer. Subsequently, 70 YTHDC1 domain crystals were manually transferred to crystallization drops for soaking and harvested (both steps were assisted by the Crystal Shifter), resulting in 30 fragment-bound structures (Bedi *et al.*, 2020 ▸). The dedicated Redissolve tab was created in the FFCS GUI on demand for this particular project, enabling the tracking of a DMSO-free soaking experiment in the FFCS DB.

In recent years great progress has been made in beamline automation, data collection and automatic data processing. This has enabled the development of dedicated xFBDD screening pipelines, which are now offered by many synchrotron facilities. The high-throughput laboratory setup combined with easy-to-use software makes more advanced early drug-discovery experiments routine for both expert and inexperienced users. At the same time, synchrotron facilities have widely expanded their services well beyond standard X-ray data collection, providing pre- and post-experiment steps such as sample preparation and data analysis, all within the same synchrotron environment.

## Supplementary Material

PDB reference: NqrF from *Pesudomonas aeruginosa*, 7qu3

PDB reference: 7qu5

PDB reference: NqrF from *Klebsiella pneumoniae*, 7qty

PDB reference: 7qu0

## Figures and Tables

**Table 1:** Success rates of FFCS campaigns for two example collaborative projects

	Project 1	Project 2
No. of crystals soaked	561 (100%)	543 (100%)
No. of crystals mounted	545 (97%)	543 (100%)
No. of crystals diffracting to ≤2.5 Å resolution	505 (90%)	412 (76%)
Data sets with *FLYNN* RSCC ≥ 0.7	41 (7.3%)	68 (12.5%)
Verified protein–fragment complexes	35 (6.2%)	35 (6.4%)

## References

[bb1] Bedi, R. K., Huang, D., Wiedmer, L., Li, Y., Dolbois, A., Wojdyla, J. A., Sharpe, M. E., Caflisch, A. & Sledz, P. (2020). *ACS Chem. Biol.* **15**, 618–625. 10.1021/acschembio.9b00894 32101404

[bb2] Cipriani, F., Röwer, M., Landret, C., Zander, U., Felisaz, F. & Márquez, J. A. (2012). *Acta Cryst.* D**68**, 1393–1399. 10.1107/S0907444912031459 22993093

[bb3] Collins, P. M., Douangamath, A., Talon, R., Dias, A., Brandao-Neto, J., Krojer, T. & von Delft, F. (2018). *Methods Enzymol.* **610**, 251–264. 10.1016/bs.mie.2018.09.027 30390801

[bb4] Collins, P. M., Ng, J. T., Talon, R., Nekrosiute, K., Krojer, T., Douangamath, A., Brandao-Neto, J., Wright, N., Pearce, N. M. & von Delft, F. (2017). *Acta Cryst.* D**73**, 246–255. 10.1107/S205979831700331X PMC5349437 28291760

[bb5] Congreve, M., Carr, R., Murray, C. & Jhoti, H. (2003). *Drug Discov. Today*, **8**, 876–877. 10.1016/s1359-6446(03)02831-9 14554012

[bb6] Davies, T. G., van Montfort, R. L., Williams, G. & Jhoti, H. (2006). *Fragment-based Approaches in Drug Discovery*, edited by W. Jahnke & D. A. Erlanson, pp. 193–214. Weinheim: Wiley-VCH.

[bb8] Douangamath, A., Powell, A., Fearon, D., Collins, P. M., Talon, R., Krojer, T., Skyner, R., Brandao-Neto, J., Dunnett, L., Dias, A., Aimon, A., Pearce, N. M., Wild, C., Gorrie-Stone, T. & von Delft, F. (2021). *J. Vis. Exp.*, e62414. 10.3791/62414 34125095

[bb9] Emsley, P., Lohkamp, B., Scott, W. G. & Cowtan, K. (2010). *Acta Cryst.* D**66**, 486–501. 10.1107/S0907444910007493 PMC2852313 20383002

[bb10] Erlanson, D. A., Fesik, S. W., Hubbard, R. E., Jahnke, W. & Jhoti, H. (2016). *Nat. Rev. Drug Discov.* **15**, 605–619. 10.1038/nrd.2016.109 27417849

[bb11] Fox, S., Farr-Jones, S., Sopchak, L., Boggs, A., Nicely, H. W., Khoury, R. & Biros, M. (2006). *J. Biomol. Screen.* **11**, 864–869. 10.1177/1087057106292473 16973922

[bb12] Hann, M. M. & Oprea, T. I. (2004). *Curr. Opin. Chem. Biol.* **8**, 255–263. 10.1016/j.cbpa.2004.04.003 15183323

[bb13] Hubbard, R. E., Davis, B., Chen, I. & Drysdale, M. J. (2007). *Curr. Top. Med. Chem.* **7**, 1568–1581. 10.2174/156802607782341109 17979768

[bb14] Huschmann, F. U., Linnik, J., Sparta, K., Ühlein, M., Wang, X., Metz, A., Schiebel, J., Heine, A., Klebe, G., Weiss, M. S. & Mueller, U. (2016). *Acta Cryst.* F**72**, 346–355. 10.1107/S2053230X16004623 PMC4854561 27139825

[bb15] Jahnke, W., Erlanson, D. A., de Esch, I. J. P., Johnson, C. N., Mortenson, P. N., Ochi, Y. & Urushima, T. (2020). *J. Med. Chem.* **63**, 15494–15507. 10.1021/acs.jmedchem.0c01608 33226222

[bb16] Koes, D. R., Baumgartner, M. P. & Camacho, C. J. (2013). *J. Chem. Inf. Model.* **53**, 1893–1904. 10.1021/ci300604z PMC3726561 23379370

[bb17] Köster, H., Craan, T., Brass, S., Herhaus, C., Zentgraf, M., Neumann, L., Heine, A. & Klebe, G. (2011). *J. Med. Chem.* **54**, 7784–7796. 10.1021/jm200642w 21972967

[bb18] Kozikowski, B. A., Burt, T. M., Tirey, D. A., Williams, L. E., Kuzmak, B. R., Stanton, D. T., Morand, K. L. & Nelson, S. L. (2003). *J. Biomol. Screen.* **8**, 210–215. 10.1177/1087057103252618 12844443

[bb19] Krojer, T., Talon, R., Pearce, N., Collins, P., Douangamath, A., Brandao-Neto, J., Dias, A., Marsden, B. & von Delft, F. (2017). *Acta Cryst.* D**73**, 267–278. 10.1107/S2059798316020234 PMC5349439 28291762

[bb20] Liebschner, D., Afonine, P. V., Baker, M. L., Bunkóczi, G., Chen, V. B., Croll, T. I., Hintze, B., Hung, L.-W., Jain, S., McCoy, A. J., Moriarty, N. W., Oeffner, R. D., Poon, B. K., Prisant, M. G., Read, R. J., Richardson, J. S., Richardson, D. C., Sammito, M. D., Sobolev, O. V., Stockwell, D. H., Terwilliger, T. C., Urzhumtsev, A. G., Videau, L. L., Williams, C. J. & Adams, P. D. (2019). *Acta Cryst.* D**75**, 861–877.

[bb21] Lima, G. M. A., Jagudin, E., Talibov, V. O., Benz, L. S., Marullo, C., Barthel, T., Wollenhaupt, J., Weiss, M. S. & Mueller, U. (2021). *Acta Cryst.* D**77**, 799–808. 10.1107/S2059798321003818 PMC8171072 34076593

[bb22] Lima, G. M. A., Talibov, V. O., Jagudin, E., Sele, C., Nyblom, M., Knecht, W., Logan, D. T., Sjögren, T. & Mueller, U. (2020). *Acta Cryst.* D**76**, 771–777. 10.1107/S205979832000889X PMC7397489 32744259

[bb23] Long, F., Nicholls, R. A., Emsley, P., Gražulis, S., Merkys, A., Vaitkus, A. & Murshudov, G. N. (2017). *Acta Cryst.* D**73**, 112–122. 10.1107/S2059798317000067 PMC5297914 28177307

[bb24] Martiel, I., Buntschu, D., Meier, N., Gobbo, A., Panepucci, E., Schneider, R., Heimgartner, P., Müller, D., Bühlmann, K., Birri, M., Kaminski, J. W., Leuenberger, J., Oliéric, V., Glettig, W. & Wang, M. (2020). *J. Synchrotron Rad.* **27**, 860–863. 10.1107/S1600577520002416 PMC7285676 32381791

[bb25] Moriarty, N. W., Grosse-Kunstleve, R. W. & Adams, P. D. (2009). *Acta Cryst.* D**65**, 1074–1080. 10.1107/S0907444909029436 PMC2748967 19770504

[bb26] Murshudov, G. N., Skubák, P., Lebedev, A. A., Pannu, N. S., Steiner, R. A., Nicholls, R. A., Winn, M. D., Long, F. & Vagin, A. A. (2011). *Acta Cryst.* D**67**, 355–367. 10.1107/S0907444911001314 PMC3069751 21460454

[bb27] Noel, J. K., Levi, M., Raghunathan, M., Lammert, H., Hayes, R. L., Onuchic, J. N. & Whitford, P. C. (2016). *PLoS Comput. Biol.* **12**, e1004794. 10.1371/journal.pcbi.1004794 PMC4786265 26963394

[bb28] Price, A. J., Howard, S. & Cons, B. D. (2017). *Essays Biochem.* **61**, 475–484. 10.1042/EBC20170029 29118094

[bb29] Schiebel, J., Krimmer, S. G., Röwer, K., Knörlein, A., Wang, X., Park, A. Y., Stieler, M., Ehrmann, F. R., Fu, K., Radeva, N., Krug, M., Huschmann, F. U., Glöckner, S., Weiss, M. S., Mueller, U., Klebe, G. & Heine, A. (2016). *Structure*, **24**, 1398–1409. 10.1016/j.str.2016.06.010 27452405

[bb30] Smart, O. S., Womack, T. O., Sharff, A., Flensburg, C., Keller, P., Paciorek, W., Vonrhein, C. & Bricogne, G. (2014). *Rhofit*. Global Phasing Ltd, Cambridge, United Kingdom.

[bb31] Spurlino, J. C. (2011). *Methods Enzymol.* **493**, 321–356. 10.1016/B978-0-12-381274-2.00013-3 21371597

[bb7] Sutanto, F., Shaabani, S., Oerlemans, R., Eris, D., Patil, P., Hadian, M., Wang, M., Sharpe, M. E., Groves, M. R. & Dömling, A. (2021). *Angew. Chem. Int. Ed.* **60**, 18231–18239. 10.1002/anie.202105584 PMC8456925 34097796

[bb32] Verlinde, C. L. M. J., Kim, H., Bernstein, B. E., Mande, S. C. & Hol, W. G. J. (1997). *Structure-Based Drug Design*, edited by P. Veerapandian, pp. 365–394. Boca Raton: CRC Press.

[bb33] Vonrhein, C., Flensburg, C., Keller, P., Sharff, A., Smart, O., Paciorek, W., Womack, T. & Bricogne, G. (2011). *Acta Cryst.* D**67**, 293–302. 10.1107/S0907444911007773 PMC3069744 21460447

[bb34] Winter, G., Waterman, D. G., Parkhurst, J. M., Brewster, A. S., Gildea, R. J., Gerstel, M., Fuentes-Montero, L., Vollmar, M., Michels-Clark, T., Young, I. D., Sauter, N. K. & Evans, G. (2018). *Acta Cryst.* D**74**, 85–97. 10.1107/S2059798317017235 PMC5947772 29533234

[bb35] Wojdyla, J. A., Kaminski, J. W., Panepucci, E., Ebner, S., Wang, X., Gabadinho, J. & Wang, M. (2018). *J. Synchrotron Rad.* **25**, 293–303. 10.1107/S1600577517014503 PMC5741135 29271779

[bb36] Wojdyr, M., Keegan, R., Winter, G. & Ashton, A. (2013). *Acta Cryst.* A**69**, s299.

[bb37] Wollenhaupt, J., Barthel, T., Lima, G. M. A., Metz, A., Wallacher, D., Jagudin, E., Huschmann, F. U., Hauss, T., Feiler, C. G., Gerlach, M., Hellmig, M., Förster, R., Steffien, M., Heine, A., Klebe, G., Mueller, U. & Weiss, M. S. (2021). *J. Vis. Exp.*, e62208. 10.3791/62208 33749678

[bb38] Wollenhaupt, J., Metz, A., Barthel, T., Lima, G. M. A., Heine, A., Mueller, U., Klebe, G. & Weiss, M. S. (2020). *Structure*, **28**, 694–706. 10.1016/j.str.2020.04.019 32413289

[bb39] Wright, N. D., Collins, P., Koekemoer, L., Krojer, T., Talon, R., Nelson, E., Ye, M., Nowak, R., Newman, J., Ng, J. T., Mitrovich, N., Wiggers, H. & von Delft, F. (2021). *Acta Cryst.* D**77**, 62–74. 10.1107/S2059798320014114 PMC7787106 33404526

